# Towards equations for estimating glomerular filtration rate without demographic characteristics

**DOI:** 10.1002/ctm2.1134

**Published:** 2022-11-30

**Authors:** Hongquan Peng, Irene Ling Ang, Xun Liu, Chiwa Aoieong, Tou Tou, Tsungyang Tsai, Kamleong Ngai, Hao I. Cheang, Peijia Liu, Terence Chuen Wai Poon

**Affiliations:** ^1^ Department of Nephrology Kiang Wu Hospital Macau China; ^2^ Pilot Laboratory Institute of Translational Medicine Centre for Precision Medicine Research and Training Faculty of Health Sciences University of Macau Macau China; ^3^ Department of Nephrology The Third Affiliated Hospital of Sun Yat‐sen University Guangzhou China; ^4^ Clinical Laboratory Kiang Wu Hospital Macau China

Dear Editor,

Estimated glomerular filtration rate (eGFR) is an essential index for identifying and monitoring chronic kidney disease (CKD). For estimating GFR, the Chronic Kidney Disease Epidemiology Collaboration (CKD‐EPI) research group recently proposed two new serum creatinine‐based equations without race, eGFRcr(AS) and eGFRcr‐cys(AS), to replace the widely used equations eGFRcr(ASR) and eGFRcr‐cys(ASR), that considers serum creatinine, cystatin C (CysC), age, sex and race.^1^, ^2^, ^3^, ^4^ The new equations were developed and validated using data mainly from White and Black participants.^4^ It remains unknown whether they are applicable to plasma samples as well as to non‐White and non‐Black subjects. The present study addressed these two questions with a focus on two Chinese CKD cohorts and showed that equations based on other blood metabolites could perform better.

The two cohorts comprised 52 patients from Guangzhou (Third Affiliated Hospital) and 135 patients from Macau (Kiang Wu Hospital) for whom serum samples and plasma samples were available, respectively (Tables S1 and S2 for their characteristics). Performance of the equations was examined with reference to the GFR measured by iohexol plasma clearance (mGFR).^2^, ^4^ The bias and precision of the current and new CKD‐EPI equations were similar for the two cohorts (Table 1). The accuracy of all the equations was better for the Macau cohort, as revealed by lower values of the root mean square error and higher values of the percent agreement within 30% of the mGFR (*P*_30_), the percent agreement within 20% of the mGFR (*P*_20_) and the percent agreement between the mGFR and eGFR categories.^5^ As plasma samples were used in the Macau cohort, our results suggested that both old and new CKD‐EPI equations should be applicable to plasma samples. In the subsequent analyses, the data from the two Chinese cohorts were pooled for a larger sample size.

**TABLE 1:** Summary of the performance of different Chronic Kidney Disease Epidemiology Collaboration (CKD‐EPI) equations for estimating the GFR in Chinese chronic kidney disease (CKD) patients using the Guangzhou cohort (serum samples) and Macau cohort (plasma samples)

Performance evaluation^a^ – equations for estimating the GFR, ml/min/1.73 m^2^ of body‐surface area			
Filtration marker and equation	*Guangzhou cohort (52 serum samples)*	*Macau cohort (135 plasma samples)*	*Overall (187 cases)*
Bias			

Mean difference (95% CI) between the mGFR and the eGFR – ml/min/1.73 m^2^ of body‐surface area			
Creatinine			
eGFRcr(ASR) [current]	6.0 (1.2–10.7)	5.3 (2.7–7.9)	5.5 (3.2–7.7)
eGFRcr(AS) [new]	7.9 (2.9–12.9)	8.5 (5.9–11.1)	8.3 (6.0–10.6)
Cystatin C			
eGFRcys(AS) [current]	.1 (−3.3–3.8)	−.6 (−2.6–1.4)	**−*.4 (*−*2.2–1.5)*** **^b^**
Creatinine‐cystatin C			
eGFRcr‐cys(ASR) [current]	2.6 (−1.3–6.8)	2.7 (.7–4.7)	***2.7 (.8–4.5)***
eGFRcr‐cys(AS) [new]	3.8 (−.2–8.0)	5.1 (3.1–7.2)	4.7 (2.8–6.6)

Median difference (95% CI) between the mGFR and the eGFR – ml/min/1.73 m^2^ of body‐surface area			
Creatinine			
eGFRcr(ASR) [current]	1.7 (−2.1–7.3)	3.6 (1.4–6.2)	3.2 (1.3–5.8)
eGFRcr(AS) [new]	3.8 (.1–9.8)	6.9 (4.4–10.1)	6.5 (3.8–9.7)
Cystatin C			
eGFRcys(AS) [current]	–.8 (–2.6–.3)	−1.4 (−3.2–.4)	**−*1.3 (*−*2.7 to* −*.02)***
Creatinine‐cystatin C			
eGFRcr‐cys(ASR) [current]	–.5 (−2.3–2.4)	2.0 (−1.2–3.7)	***.8 (*−*.7 to 2.6)***
eGFRcr‐cys(AS) [new]	.4 (−1.4–3.2)	3.9 (1.6–6.3)	2.2 (1.0–4.5)
Precision			

Interquartile range (IQR) of the difference (95% CI) between the mGFR and the eGFR ml/min/1.73 m^2^ of body‐surface area			
Creatinine			
eGFRcr(ASR) [current]	22.5 (11.9–26.3)	16.5 (12.7–20.2)	18.3 (14.3–21.3)
eGFRcr(AS) [new]	24.4 (13.5–28.1)	19.2 (13.5–22.0)	19.9 (16.5–23.1)
Cystatin C			
eGFRcys(AS) [current]	8.7 (5.4–21.7)	12.9 (10.6–15.7)	***11.4 (9.4–14.6)***
Creatinine–cystatin C			
eGFRcr‐cys(ASR) [current]	13.9 (7.2–25.2)	13.6 (10.3–17.1)	***13.8 (11.0–17.0)***
eGFRcr‐cys(AS) [new]	15.4 (7.4–26.8)	14.1 (11.5–17.9)	15.0 (12.2–18.1)
Accuracy^c^			

Root mean square error (RMSE) relative to the mGFR (95% CI) – %			
Creatinine			
eGFRcr(ASR) [current]	39.3 (29.1–50.1)	34.5 (27.7–41.1)	35.9 (30.0–41.8)
eGFRcr(AS) [new]	40.8 (29.8–52.7)	39.2 (31.9–46.5)	39.6 (33.3–46.1)
Cystatin C			
eGFRcys(AS) [current]	29.2 (21.0–36.9)	23.3 (18.9–27.5)	***25.1 (21.2–28.9)***
Creatinine‐cystatin C			
eGFRcr‐cys(ASR) [current]	31.5 (22.3–40.8)	25.7 (20.7–30.7)	***27.5 (22.8–32.0)***
eGFRcr‐cys(AS) [new]	31.7 (22.2–41.6)	28.0 (22.5–33.3)	29.1 (24.2–33.9)

Percent agreement (95% CI) within 30% of the mGFR, *P*_30_ – %			
Creatinine			
eGFRcr(ASR) [current]	62 (48–75)	79 (73–86)	74 (68–80)
eGFRcr(AS) [new]	60 (46–73)	73 (65–80)	69 (62–75)
Cystatin C			
eGFRcys(AS) [current]	77 (65–87)	87 (81–93)	***84 (79–89)***
Creatinine‐cystatin C			
eGFRcr‐cys(ASR) [current]	71 (58–83)	87 (81–93)	***82 (76–88)***
eGFRcr‐cys(AS) [new]	65 (52–77)	84 (78–90)	79 (73–85)

Percent agreement (95% CI) within 20% of the mGFR, *P*_20_ – %			
Creatinine			
eGFRcr(ASR) [current]	40 (27–54)	61 (53–69)	55 (48–62)
eGFRcr(AS) [new]	35 (23–48)	53 (45–62)	48 (41–55)
Cystatin C			
eGFRcys(AS) [current]	56 (42–69)	73 (65–80)	***68 (61–75)***
Creatinine–cystatin C			
eGFRcr‐cys(ASR) [current]	52 (38–65)	70 (63–78)	***65 (58–72)***
eGFRcr‐cys(AS) [new]	52 (38–65)	68 (61–76)	64 (57–71)
Correct classification			

Percent agreement (95% CI) between the mGFR and eGFR categories – %			
Creatinine			
eGFRcr(ASR) [current]	60 (46–73)	70 (62–78)	67 (60–74)
eGFRcr(AS) [new]	52 (37–65)	64 (56–73)	61 (54–68)
Cystatin C			
eGFRcys(AS) [current]	65 (52–79)	73 (66–81)	***71 (65–78)***
Creatinine–cystatin C			
eGFRcr‐cys(ASR) [current]	67 (54–79)	74 (66–81)	***72 (66–78)***
eGFRcr‐cys(AS) [new]	62 (48–73)	75 (67–81)	***71 (64–78)***

Abbreviations: eGFR, estimated glomerular filtration rate; mGFR, measured glomerular filtration rate.

^a^ The performance of each equation in estimating the GFR was evaluated in terms of bias, precision and accuracy, according to the methods described by Inker et al. (2012)^2^ and Inker et al. (2021)^4^.

^b^ The values indicating the best two equations were italicized and made bold.

^c^ Accuracy was calculated as the RMSE relative to the mGFR, the percentage of estimates that differed from the mGFR by less than 30% (*P*_30_), and the percentage that differed by less than 20% (*P*_20_).

A *P*_30_ value of 80%–90% is considered acceptable, and a *P*_30_ value of 90% or higher is preferred.^4^ However, *P*_30_ values of two new equations without race were less than 80% for the two Chinese cohorts (Table 1, Result S1 for statistical test results). LOWESS curve fitting of the agreement between the mGFR and eGFR indicated that all the serum creatinine‐based equations overestimated the GFR in the range of 30–90 ml/min/1.73 m^2^ (Figure 1). Overestimation was the highest for the eGFRcr(AS) equation (Figure 1B), whereas it was not obviously for the eGFRcys(AS) equation (Figure 1E). In a recent study, applying the eGFRcr(AS) equation to the White European population also resulted in shifting a major proportion of CKD patients to a higher eGFR category.^6^ In addition to the unsatisfactory *P*_30_ values, the use of the new equations could lead to underestimation of the disease severity in Chinese patients with mild or moderate loss of kidney function. Therefore, we recommend not to use the new equations without race for Chinese CKD patients.

**FIGURE 1:**
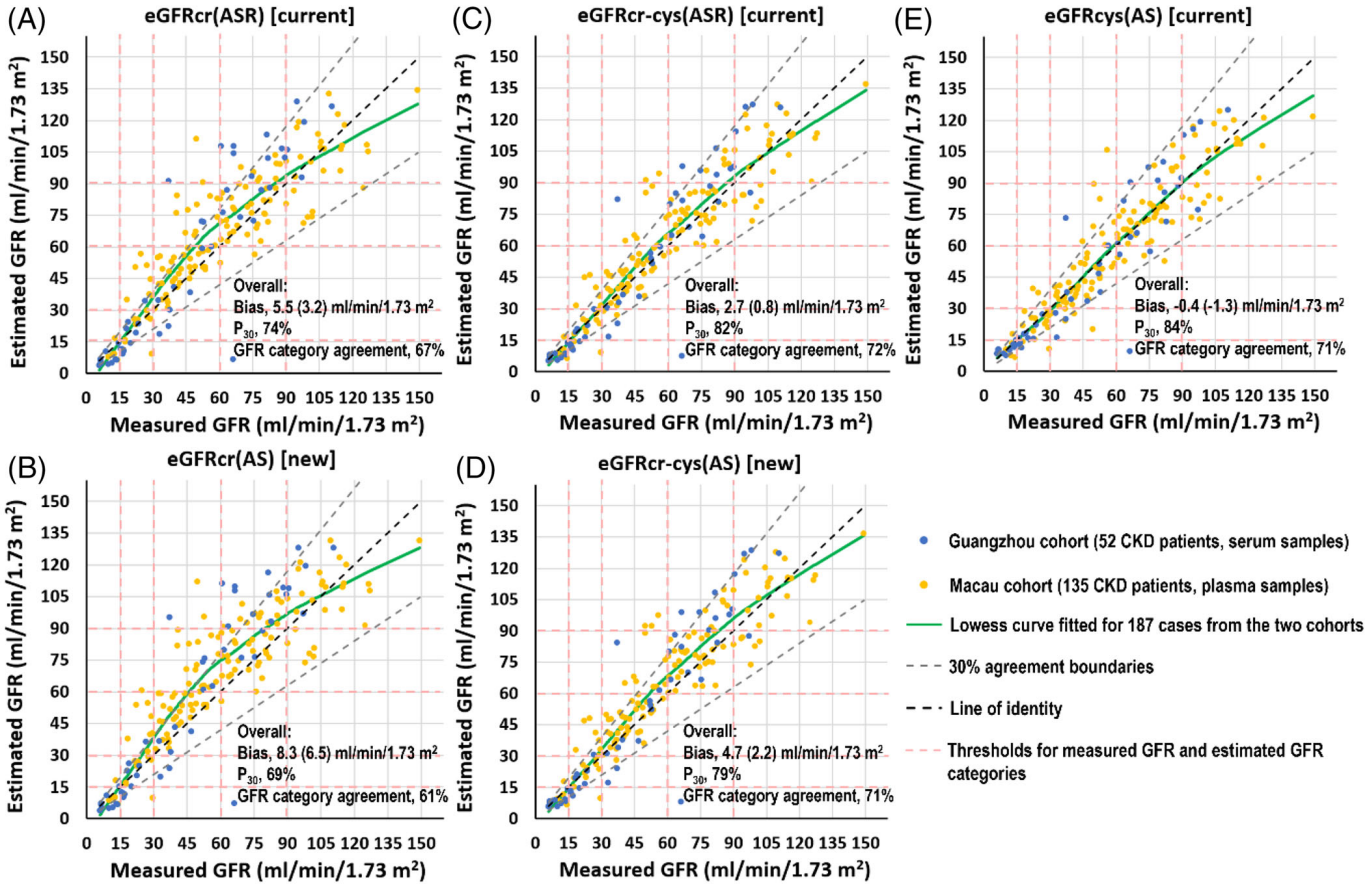
Comparison of the measured glomerular filtration rate (mGFR) and estimated glomerular filtration rate (eGFR) for alternative GFR estimating equations in the two Chinese cohorts. The Chronic Kidney Disease Epidemiology Collaboration (CKD‐EPI) equations for estimating the GFR are named according to the name provided by Inker et al.^4^ They are referred to by the filtration marker or markers (creatinine [eGFRcr], cystatin C [eGFRcys] or creatinine–cystatin C [eGFRcr‐cys]) and the demographic factors (age, sex and race [ASR] or age and sex [AS]) that were used in their development. They are (A) eGFRcr(ASR), (B) eGFRcr(AS), (C) eGFRcr‐cys(ASR), (D) eGFRcr‐cys(AS), and (E) eGFRcys(AS). Data from the two Chinese chronic kidney disease (CKD) cohorts (Guangzhou cohort and Macau cohort) are shown. Overall, we referred to 185 CKD patients from the 2 cohorts. Bias was defined as the mean difference (median difference) between the mGFR and eGFR. A positive sign indicates an overestimation of the mGFR, and a negative sign indicates an underestimation of the mGFR. *P*_30_ is the percent agreement within 30% of the mGFR. GFR category agreement is the percent agreement between the mGFR and eGFR categories (<15, 15–29, 30–59, 60–89 and ≥90 ml/min/1.73 m^2^). For each plot, the LOWESS curve was fitted using the data from 185 CKD patients.

To minimize the effect of systemic and systematic biases,^6^, ^7^ to ensure a metabolite marker can be applied to both serum and plasma specimens,^8^ and to avoid false‐positive biomarkers,^9^ we adopted a stringent two‐centre study design (Figure 2), involving plasma samples from 10 healthy volunteers in addition to the two Chinese cohorts (Table S3, Discussion S1 about study designs). The samples were subjected to untargeted metabolomics profiling using a Metabolon's Discovery HD4 platform.^10^ The final list of putative biomarkers contained 212 metabolites (207 negatively and 5 positively correlated with the mGFR, Figure 2 and Result S2 for details). Among the top 20 putative biomarkers (Table S5), hydroxyasparagine and *N,N*‐dimethyl‐proline–proline had not been previously shown to be inversely associated with kidney function (Table S6, Discussion S2 about accessibility in clinical practice). Furthermore, one of the top 20 putative biomarkers was creatinine, indicating the success of our study design.

**FIGURE 2:**
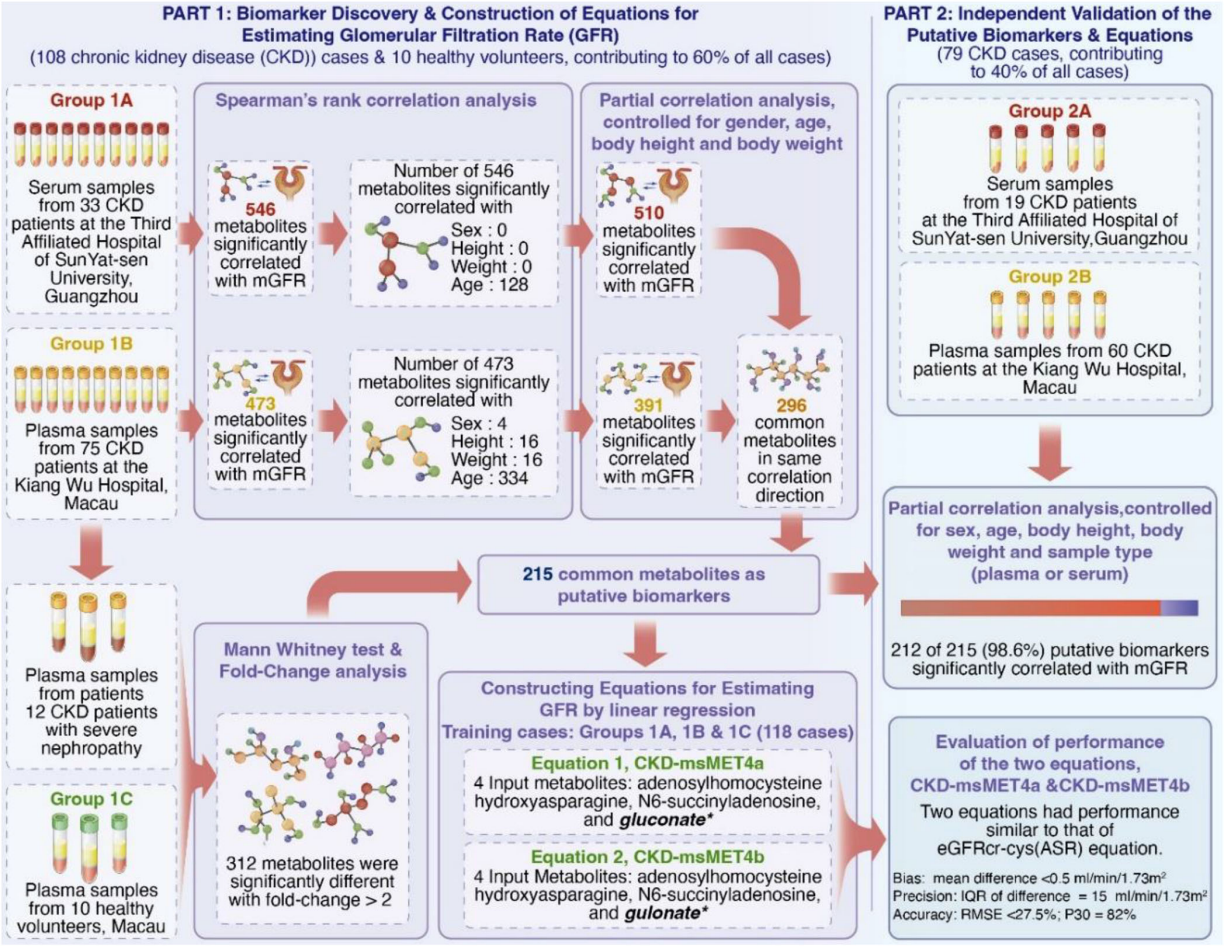
Schematic illustration of the two‐centre study design and participant flow for discovery and validation of the metabolite markers for estimating the GFR in patients with chronic kidney disease (CKD). The study design comprised two parts. Part 1 was for biomarker discovery and construction of equations for estimating the GFR, whereas Part 2 was for independent validation of the putative biomarkers and equations. The study design involved (i) two types of blood samples, serum and plasma, from two centres and (ii) plasma samples from healthy volunteers to minimize false discovery of biomarkers associated with systemic and/or systematic bias(es). The serum samples were collected from 52 Chinese CKD patients at the Third Affiliated Hospital of Sun Yat‐sen University in Guangzhou; the plasma samples were collected from 135 Chinese CKD patients at Kiang Wu Hospital and 10 healthy volunteers in Macau, resulting in a total of 197 subjects. Seventy‐nine CKD patients (19 CKD patients from Guangzhou and 60 CKD patients from Macau, accounting for 40% of all the participants) were randomly selected to form an independent validation group. The rest of the participants were assigned for biomarker discovery and for construction of equations to estimate the GFR. The statistical methods used at different steps and the summary of the corresponding results are also indicated in the figure. Before the partial correlation analysis and equation construction, the measured glomerular filtration rate (mGFR), normalized abundances of the putative metabolite markers, serum/plasma creatinine level and serum/plasma cystatin‐C (cysC) level were logarithmically transformed. Top 20 candidates of 215 putative biomarkers (pseudouridine, *N*‐acetylserine, C‐glycosyltryptophan, 3‐(3‐amino‐3‐carboxypropyl)uridine, 5,6‐dihydrouridine, hydroxyasparagine, N6‐carbamoylthreonyladenosine, *S*‐adenosylhomocysteine, gulonate, 2,3‐dihydroxy‐5‐methylthio‐4‐pentenoate, creatinine, *N*‐acetylneuraminate, *N,N*‐dimethyl‐proline–proline, erythronate, N6‐succinyladenosine, *N*‐acetylalanine, *O*‐sulfo‐l‐tyrosine, arabonate/xylonate, 4‐acetamidobutanoate and gluconate) were used to construct equations for estimating GFR. Equation 1, CKD‐msMET4a is as follows: estimated glomerular filtration rate (eGFR) = exp(3.999 − .137 ln(normalized abundance of *S*‐adenosylhomocysteine) − .162 ln(normalized abundance of gluconate) − .209 ln(normalized abundance of N6‐succinyladenosine) − .319 ln(normalized abundance of hydroxyasparagine)). Equation 2, CKD‐msMET4b is as follows: eGFR = exp(3.991 − .141 ln(normalized abundance of *S*‐adenosylhomocysteine) − .146 ln(normalized abundance of gulonate) − .214 ln(normalized abundance of N6‐succinyladenosine) − .270 ln(normalized abundance of hydroxyasparagine)). *Gluconate and gulonate are stereoisomers of each other, indicating that the two equations, CKD‐msMET4a and CKD‐msMET4b, are almost identical in nature. The two equations have performance similar to that of the current Chronic Kidney Disease Epidemiology Collaboration (CKD‐EPI) creatinine‐CysC equation with race, that is eGFRcr‐cys(ASR).

For proof‐of‐concept, we attempted to construct eGFR equations from the data of the top 20 putative biomarkers in the discovery dataset. *S*‐Adenosylhomocysteine, gluconate, N6‐succinyladenosine and hydroxyasparagine (*p* values <.05) were retained in the final regression equation named CKD‐msMET4a (Table 2). As gluconate is a common component of food additives and supplements, the performance of CKD‐msMET4a could be affected by gluconate intake. We hence constructed another equation named CKD‐msMET4b with the exclusion of gluconate. The major difference from the first equation was the replacement of gluconate with gulonate (Result S3 for additional information). Gulonate and gluconate are stereoisomers of each other, indicating that the two equations are almost identical in nature. Further inclusion of age, sex, height, weight and/or serum/plasma CysC did not significantly improve the two equations (*p* values >.05).

**TABLE 2:** Summary of the performance of different equations for estimating the GFR in the independent validation group^a^

Performance evaluation^b^ – equations for estimating the GFR, ml/min/1.73 m^2^ of body‐surface area	
Bias	

Mean difference (95% CI) between the mGFR and the eGFR – ml/min/1.73 m^2^ of body‐surface area	
CKD‐msMET4a^d^	***.3 (−2.5–3.0)* ^c^**
CKD‐msMET4b^e^	***−.3 (−3.0–2.4)***
eGFRcr(ASR) [current]	4.7 (1.1–8.3)
eGFRcr(AS) [new]	7.7 (4.1–11.4)
eGFRcys(AS) [current]	−.8 (−3.5–2.1)
eGFRcr‐cys(ASR) [current]	2.1 (−.7–4.9)
eGFRcr‐cys(AS) [new]	4.3 (1.5–7.2)

Median difference (95% CI) between the mGFR and the eGFR – ml/min/1.73 m^2^ of body‐surface area	
CKD‐msMET4a	1.3 (−3.5–4.9)
CKD‐msMET4b	***.9 (−3.1–3.6)***
eGFRcr(ASR) [current]	3.5 (−.7–7.6)
eGFRcr(AS) [new]	6.5 (2.9–10.1)
eGFRcys(AS) [current]	−2.0 (−4.3–2.2)
eGFRcr‐cys(ASR) [current]	***.9 (−2.0 to 3.7)***
eGFRcr‐cys(AS) [new]	3.3 (−.1–5.7)
Precision	

Interquartile range (IQR) of the difference (95% CI) between the mGFR and the eGFR – ml/min/1.73 m^2^ of body‐surface area	
CKD‐msMET4a	***14.5 (11.8–19.4)***
CKD‐msMET4b	***14.6 (10.4–20.0)***
eGFRcr(ASR) [current]	19.8 (14.0–23.5)
eGFRcr(AS) [new]	20.8 (15.0–24.6)
eGFRcys(AS) [current]	15.4 (10.3–21.2)
eGFRcr‐cys(ASR) [current]	15.6 (11.7–21.8)
eGFRcr‐cys(AS) [new]	17.6 (12.5–22.3)
Accuracy^f^	

Root mean square error (RMSE) relative to the mGFR (95% CI) – %	
CKD‐msMET4a	27.2 (21.6–33.0)
CKD‐msMET4b	***26.3 (20.9–31.7)***
eGFRcr(ASR) [current]	36.0 (27.1–45.5)
eGFRcr(AS) [new]	40.0 (30.3–50.4)
eGFRcys(AS) [current]	***25.2 (19.9–31.5)***
eGFRcr‐cys(ASR) [current]	27.2 (20.1–35.2)
eGFRcr‐cys(AS) [new]	28.9 (21.5–37.3)

Percent agreement (95% CI) within 30% of the mGFR, *P*_30_ – %	
CKD‐msMET4a	82 (73–91)
CKD‐msMET4b	82 (73–91)
eGFRcr(ASR) [current]	73 (63–82)
eGFRcr(AS) [new]	70 (58–80)
eGFRcys(AS) [current]	***84 (75–91)***
eGFRcr‐cys(ASR) [current]	***85 (76–92)***
eGFRcr‐cys(AS) [new]	80 (71–89)

Percent agreement (95% CI) within 20% of the mGFR, *P*_20_ – %	
CKD‐msMET4a	62 (51–73)
CKD‐msMET4b	***65 (54–76)***
eGFRcr(ASR) [current]	56 (44–67)
eGFRcr(AS) [new]	44 (34–56)
eGFRcys(AS) [current]	61 (49–72)
eGFRcr‐cys(ASR) [current]	61 (49–71)
eGFRcr‐cys(AS) [new]	***63 (52–75)***
Correct classification	

Percent agreement (95% CI) between the mGFR and eGFR categories – %	
CKD‐msMET4a	***70 (59–80)***
CKD‐msMET4b	***70 (59–80)***
eGFRcr(ASR) [current]	68 (57–78)
eGFRcr(AS) [new]	65 (53–75)
eGFRcys(AS) [current]	63 (52–73)
eGFRcr‐cys(ASR) [current]	***70 (59–80)***
eGFRcr‐cys(AS) [new]	68 (58–78)

Abbreviations: CKD, chronic kidney disease; eGFR, estimated glomerular filtration rate; mGFR, measured glomerular filtration rate.

^a^ Independent validation cases included serum samples from 19 CKD patients, the Third Affiliated Hospital of Sun Yat‐Sen University, Guangzhou and plasma samples from 60 CKD patients, Kiang Wu Hospital, Macau.

^b^ The performance of each equation in estimating the GFR was evaluated in terms of bias, precision and accuracy, according to the methods described by Inker et al. (2012)^2^ and Inker et al. (2021)^4^.

^c^ Although the statistical power was insufficient, the values suggesting the best two equations were italicized and made bold

^d^ CKD‐msMET4a equation, eGFR = exp(3.999 − .137 ln(normalized abundance of *S*‐adenosylhomocysteine) − .162ln(normalized abundance of gluconate) − .209 ln(normalized abundance of N6‐succinyladenosine) − .319 ln(normalized abundance of hydroxyasparagine)).

^e^ CKD‐msMET4b equation, eGFR = exp(3.991 − .141 ln(normalized abundance of *S*‐adenosylhomocysteine) − .146 ln(normalized abundance of gulonate) − .214 ln(normalized abundance of N6‐succinyladenosine) − .270 ln(normalized abundance of hydroxyasparagine)).

^f^ Accuracy was calculated as the RMSE relative to the mGFR, the percentage of estimates that differed from the measured GFR by less than 30% (*P*_30_), and the percentage that differed by less than 20% (*P*_20_).

Using the data from the independent validation cases, the performance of the two equations was compared with that of the CKD‐EPI eGFR equations (Figure 2). Concerning bias, precision, accuracy and GFR category agreement, either CKD‐msMET4a or CKD‐msMET4b equations appeared to be one of the best two equations although the statistical power was insufficient (Table 2, Result S4 for detailed results). The *P*_30_ values of both equations were 82%. They were not significantly different from those of the current eGFRcr‐cys(ASR) equation (*p*‐values >.890) but were significantly different from the *P*_30_ value of the new eGFRcr(AS) equation (*p*‐values <.05). LOWESS curves revealed that the overestimation of GFR in the range of 30–90 ml/min/1.73 m^2^ was not observed for the CKD‐msMET4a and CKD‐msMET4b equations (Figure S1, Result S4 for detailed results).

Although the male and female patients might have differences in age and total mass muscles, GFRs estimated by the two equations were not significantly different between the male and female patients (*p*‐values <.005, Table S7, Result S4 for detailed results). This suggests that four metabolites could provide sufficient information for estimating the GFR without demographic characteristics. This may also suggest that these two equations are applicable to patients of other races, such as the White and Black populations.

In conclusion, the new CKD‐EPI creatinine‐based equations without race could lead to underestimation of the disease severity in Chinese patients with mild or moderate loss of kidney function. However, our results suggest the possibility of developing new metabolite equations for estimating the GFR without demographic characteristics.

## CONFLICT OF INTEREST

The authors declare no conflict of interest.

## Supporting information

Supporting Information

Supporting Information

## Data Availability

The datasets used and analysed during the current study are available from the corresponding authors on reasonable request.
