# Supplementary material for: Towards equations for estimating glomerular filtration rate without demographic characteristics
Source: Clin Transl Med. 2022 Nov 30;12(12):e1134. doi: 10.1002/ctm2.1134 (PMC9709889; doi:10.1002/ctm2.1134)
Supplement: Supplementary file 1 — Supporting Information [file CTM2-12-e1134-s002.docx]

**Toward Equations For Estimating Glomerular Filtration Rate without Demographic Characteristics**

**Running title: Toward eGFR Equations without Demographics**

**Hongquan Peng^1,^*, Irene Ling Ang^2^, Xun Liu^3^, Chiwa Aoieong^1^, Tou Tou^1^, Tsungyang Tsai^1^, Kamleong Ngai^4^, Hao I Cheang^4^, Peijia Liu^3^, Terence Chuen Wai Poon^2^**

**Affiliations:**

^1^Department of Nephrology, Kiang Wu Hospital, Macau, China;

^2^Pilot Laboratory, Institute of Translational Medicine, Centre for Precision Medicine Research and Training, Faculty of Health Sciences, University of Macau, Macau, China;

^3^Department of Nephrology, The Third Affiliated Hospital of Sun Yat-sen University, Guangzhou, China;

^4^Clinical Laboratory, Kiang Wu Hospital, Macau, China.

**Supporting Information:**

Supplemental Methods

Supplemental Results S1 to S4

Supplemental Discussions S1 & S2

Supplemental Tables S1 to S7

Supplemental Figure S1

Supplemental References

**SUPPLEMENTAL METHODS**

***Study Participants and Specimens***

A total of 197 participants (96 females, 48.7%), namely, 10 healthy volunteers and 187 chronic kidney disease (CKD) patients with varying degrees of renal dysfunction, were enrolled in this study; 52 CKD patients were from the Third Affiliated Hospital of Sun Yat-sen University in Guangzhou, 135 CKD patients and 10 healthy volunteers were from Kiang Wu Hospital in Macau. The inclusion criteria were as follows: 1) age >18 years and 2) healthy volunteers without acute/chronic diseases and voluntary CKD patients. The exclusion criteria were as follows: 1) acute kidney injury; 2) dehydration, congestive heart failure, obvious peripheral edema, and other severe fluid balance disorders; 3) physical disability and skeletal muscle atrophy; 4) urinary tract obstruction; 5) patients who had recently taken the following drugs and could not suspend their use: aspirin, nonsteroidal anti-inflammatory drugs, cimetidine, ranitidine and others; 6) an allergy to iodine contrast agents; 7) presence of thyroid disease; 8) pregnant or breastfeeding; 9) cancer patients; and 10) dialysis patients. The glomerular filtration rate (GFR) of the individual participants was measured by iohexol plasma clearance. Serum samples were collected from the CKD patients from the Third Affiliated Hospital, while plasma samples were collected from the CKD patients from Kiang Wu Hospital and the healthy volunteers. All the samples were stored at -80°C for this study. The study protocol was approved by the institutional review board (i.e., the ethics committee) of Kiang Wu Hospital (KWH 2018–001). All methods were carried out in accordance with relevant guidelines and regulations. All the participants signed a written informed consent form.

***CKD-EPI Equations for Estimating the GFR***

Three current CKD-EPI equations and two new CKD-EPI equations that do not consider race were used in the present study. Their coefficients are according to the values reported by Inker et al.^4^ The equations are provided as follows.

Equation 1. 2009 CKD-EPI creatinine, eGFRcr(ASR), current

eGFR = 141 × min(SCr/κ,1)^α^ × max(SCr/κ,1)^-1.209^ × 0.9929^Age^ × (1.018 if female) × (1.159 if black)

where eGFR in mL/min/1.73 m^2^

SCr (standardized serum creatinine) in mg/dL (i.e., μmol/L/88.42)

κ = 0.7 (females) or 0.9 (males)

α = -0.329 (females) or -0.411 (males)

min = indicates the minimum of SCr/κ or 1

max = indicates the maximum of SCr/κ or 1

age = years

Equation 2. 2012 CKD-EPI creatinine-cystatin C, eGFRcr-cys(ASR), current

eGFR = 135 × min(SCr/κ,1)^α^ × max(SCr/κ,1)^-0.601^ × min(Scys/0.8,1)^-0.375^ × max(Scys/0.8,1)^-0.711^ × 0.9952^Age^ × (0.969 if female) × (1.08 if black)

where eGFR in mL/min/1.73 m^2^

SCr in mg/dL (i.e., µmol/L/88.42)

Scys (standardized serum cystatin C) in mg/L

κ = 0.7 (females) or 0.9 (males)

α = -0.248 (females) or -0.207 (males)

min(SCr/κ or 1) = indicates the minimum of SCr/κ or 1

max(SCr/κ or 1) = indicates the maximum of SCr/κ or 1

min(Scys/0.8, 1) = indicates the minimum of Scys/0.8, 1

max(Scys/0.8, 1) = indicates the maximum of Scys/0.8, 1

age = years

Equation 3. 2012 CKD-EPI Cystatin C, eGFRcys(AS), current

eGFR = 133 × min(Scys/0.8,1)^-0.499^ × max (Scys/0.8, 1)^-1.328^ × 0.9962^Age^ × (0.932 if female)

where eGFR = mL/min/1.73 m^2^

Scys = mg/L

min = indicates the minimum of Scys/0.8 or 1

max = indicates the maximum of Scys/0.8 or 1

age = years

Equation 4. 2021 CKD-EPI creatinine, eGFRcr(AS), new

eGFR = 142 × min(SCr/κ,1)^α^ × max(SCr/κ,1)^-1.200^ × 0.9938^Age^ × (1.012 if female)

where eGFR in mL/min/1.73 m^2^

SCr in mg/dL (i.e., umol/L/88.42)

κ = 0.7 (females) or 0.9 (males)

α = -0.241 (females) or -0.302 (males)

min = indicates the minimum of SCr/κ or 1

max = indicates the maximum of SCr/κ or 1

age = years

Equation 5. 2021 CKD-EPI creatinine-cystatin C, eGFRcr-cys(AS), new

eGFR = 135 × min(SCr/κ,1)^α^ × max(SCr/κ,1)^-0.544^ × min(Scys/0.8,1)^-0.323^ × max(Scys/0.8,1)^-0.778^ × 0.9961^Age^ × (0.963 if female)

where eGFR in mL/min/1.73 m^2^

SCr in mg/dL (i.e., umol/L/88.42)

Scys (standardized serum cystatin C) in mg/L

κ = 0.7 (females) or 0.9 (males)

α = -0.219 (females) or -0.144 (males)

min(SCr/κ or 1) = indicates the minimum of SCr/κ or 1

max(SCr/κ or 1) = indicates the maximum of SCr/κ or 1

min(Scys/0.8, 1) = indicates the minimum of Scys/0.8, 1

max(Scys/0.8, 1) = indicates the maximum of Scys/0.8, 1

age = years

***Study Design for Identifying and Validating Metabolite Markers for Estimating the GFR***

The study design of the present study is summarized in Figure 1 in the main manuscript. The study comprised two major parts: Part 1: biomarker discovery and construction of equations for estimating the GFR, and Part 2: independent validation of the putative biomarkers and equations. The 187 CKD patients were randomly separated into two major groups: one major group for the discovery of biomarkers and construction of equations for estimating the GFR (33 CKD patients from Guangzhou, Group 1A, and 75 CKD patients from Macau, Group 1B, accounting for 55% of all participants) and one major group for independent validation of the putative biomarkers and the equations (Group 2A: 19 CKD patients from Guangzhou; Group 2B: 60 CKD patients from Macau, accounting for 40% of all participants). The 10 healthy volunteers (Group 1C, contributing to 5% of all participants) were also assigned to the discovery of biomarkers and construction of equations.

In Part 1, metabolites significantly associated with the measured GFR (mGFR) were first identified in serum samples of Group 1A and plasma samples of Group 1B. Metabolites that were significantly correlated with the mGFR in the same direction in both groups were retained. In Group 1B, there were 12 CKD patients with severe nephropathy (mGFR <30 mL/min per 1.73 m^2^). To avoid the discovery of false biomarkers, we further compared the abundances of the metabolites in the plasma samples between the 12 CKD patients with severe nephropathy (from Discovery Group 1B) and the 10 healthy volunteers (Discovery Group 1C). Only those significantly different between the two groups, with a fold change >2 in a direction consistent with the correlation analysis results, were retained as putative biomarkers.^9^ The putative biomarkers were ranked according to the degrees of their inverse associations with the mGFR. The serum/plasma abundances of the top 20 biomarkers (having the highest absolute values of correlation coefficient) were subjected to the construction of equations for estimating the GFR. In Part 2, cases from Group 2A and Group 2B (a total of 79 CKD cases) were combined and used for independent validation of the individual putative biomarkers and the equations for estimating the GFR.

***Metabolomics Analysis***

***Untargeted Metabolomics Profiling Platform***

Untargeted metabolomics profiling was performed using the Discovery HD4™ platform (Metabolon, Morrisville, NC, USA), a well-established platform that allows unambiguous identification and precise quantification of metabolites in biological specimens. It was launched in 2014. The platform has been successfully applied to various metabolomics studies.^10-12^ In the present study, untargeted metabolomics analyses of the samples were conducted at the Calibra-Metabolon Joint Laboratory (Hangzhou, China).

***Sample Accession***

Each sample was added to the mLIMS system and was assigned by the LIMS a unique identifier that was associated with the original source identifier only. This identifier was used to track all sample handling, tasks, the results, etc. The samples (and all derived aliquots) were tracked by the LIMS system. All portions of any sample were automatically assigned their own unique identifiers by the LIMS when a new task was created; the relationship of these samples was also tracked. All the samples were maintained at -80°C until processed.

***Sample Preparation***

The samples were prepared using the automated MicroLab STAR^®^ system from Hamilton Company. Several recovery standards were added prior to the first step in the extraction process for QC purposes. To remove proteins, dissociate small molecules bound to proteins or trapped in the precipitated protein matrix, and recover chemically diverse metabolites, proteins were precipitated with methanol under vigorous shaking for 2 min (Glen Mills GenoGrinder 2000) followed by centrifugation.

***Quality Assurance and Quality Control***

Several types of controls were analyzed in concert with the experimental samples: a pooled matrix sample generated by taking a small volume of each experimental sample (or alternatively, use of a pool of well-characterized human plasma) served as a technical replicate throughout the dataset; extracted water samples served as process blanks; and a cocktail of QC standards that were carefully chosen not to interfere with the measurement of endogenous compounds were spiked into every analyzed sample, allowed instrument performance monitoring and aided chromatographic alignment. Instrument variability was determined by calculating the median relative standard deviation (RSD) for the standards added to each sample prior to injection into the mass spectrometers. Overall process variability was determined by calculating the median RSD for all endogenous metabolites (i.e., non-instrument standards) present in 100% of the pooled matrix samples. Experimental samples were randomized across the platform run with QC samples spaced evenly among the injections.

***UPLC–MS/MS Methods***

Ultra-performance liquid chromatography–tandem mass spectrometry (UPLC–MS/MS) was performed using a Waters ACQUITY UPLC and a Thermo Scientific Q-Exactive high resolution/accurate mass spectrometer interfaced with a heated electrospray ionization (HESI-II) source and Orbitrap mass analyzer operated at 35,000 mass resolution. Each sample extract was divided into five fractions: two for analysis by two separate reverse-phase (RP)/UPLC–MS/MS methods with positive ion mode electrospray ionization (ESI), one for analysis by RP/UPLC–MS/MS with negative ion mode ESI, one for analysis by HILIC/UPLC–MS/MS with negative ion mode ESI, and one sample was reserved for backup. Samples were placed briefly on a TurboVap® (Zymark) to remove the organic solvent. The sample extracts were stored overnight under nitrogen before preparation for analysis. The sample extract was dried and then reconstituted in solvents compatible with each of the four methods. Each reconstitution solvent contained a series of standards at fixed concentrations to ensure injection and chromatographic consistency. One aliquot was analyzed using acidic positive ion conditions and was chromatographically optimized for more hydrophilic compounds. In this method, the extract was gradient eluted from a C18 column (Waters UPLC BEH C18-2.1x100 mm, 1.7 µm) using water and methanol, containing 0.05% perfluoropentanoic acid (PFPA) and 0.1% formic acid (FA). Another aliquot was also analyzed using acidic positive ion conditions, which were chromatographically optimized for more hydrophobic compounds. In this method, the extract was gradient eluted from the same aforementioned C18 column using methanol, acetonitrile, water, 0.05% PFPA and 0.01% FA and was operated at an overall higher organic content. Another aliquot was analyzed using basic negative ion optimized conditions using a separate dedicated C18 column. The basic extracts were gradient eluted from the column using methanol and water but with 6.5 mM ammonium bicarbonate at pH 8. The fourth aliquot was analyzed via negative ionization following elution from a HILIC column (Waters UPLC BEH amide 2.1x150 mm, 1.7 µm) using a gradient consisting of water and acetonitrile with 10 mM ammonium formate, pH 10.8. The MS analysis alternated between MS and data-dependent MSn scans using dynamic exclusion. The scan range varied slightly between methods but covered 70-1000 m/z.

***Data Extraction and Metabolite Identification***

The raw data were extracted, peak-identified and QC processed using Discovery HD4 hardware and software. These systems are built on a web-service platform utilizing Microsoft. NET technologies run on high-performance application servers and fiber-channel storage arrays in clusters to provide active failover and load balancing. Compounds were identified by comparison to library entries of purified standards or recurrent unknown entities.

***Discovery HD4***

Discovery HD4 maintains a library based on authenticated standards that contain the retention time/index (RI), mass to charge ratio (m/z), and chromatographic data (including MS/MS spectral data) on all molecules present in the library. Furthermore, biochemical identifications are based on three criteria: retention index within a narrow RI window of the proposed identification, accurate mass match to the library +/- 10 ppm, and the MS/MS forward and reverse scores between the experimental data and authentic standards. The MS/MS scores are based on a comparison of the ions present in the experimental spectrum to the ions present in the library spectrum.

***Metabolite Quantification and Data Normalization***

The MS peaks of individual metabolites were quantified using the area under the curve. For studies spanning multiple days, a data normalization step was performed to correct variation resulting from instrument interday tuning differences. Essentially, each compound was corrected in run-day blocks by registering the medians to equal one (1.00) and normalizing each data point proportionately (termed the block correction).

***Construction of Equations for Estimating GFR***

We attempted to construct eGFR equations from the data of the top 20 putative biomarkers in the discovery dataset by undertaking the approach described by the CKD-EPI research group. ^4^ Briefly, multiple linear regression (stepwise backward elimination) was applied to relate ln(mGFR) to natural logarithm-transformed normalized abundances of the top 20 putative metabolite markers in the discovery dataset. This aimed to identify and only retain metabolite markers with a statistically significant independent contribution to GFR estimation.

***Evaluation of Equation Performance***

Evaluation of equation performance was performed as previously described by the CKD-EPI research group.^2,4^ Bias was assessed as the mean of the difference and the median of the difference between the mGFR and the eGFR. Precision was assessed as the interquartile range for the difference. Accuracy was assessed as the root mean square error relative to the mGFR, the percent of estimates within 30% of the mGFR (P_30_), the percent of estimates within 20% of the mGFR (P_20_), and the percent agreement between the mGFR and eGFR categories^5^ (<15, 15–29, 30–59, 60–89, ≥90 ml/min/1.73 m2). The 95% confidence intervals were obtained by running 2000 bootstraps.

***Statistical Analyses***

ANCOVA (analysis of covariance) was used to compare the natural log-transformed blood levels of creatinine and CysC between male and female CKD patients with age and natural log-transformed mGFR as covariates. ANCOVA was also used to compare the natural log-transformed eGFRs between male and female CKD patients with natural log-transformed mGFR as covariates. One proportion z-test (two-tailed) was used to compare the P_30_ value of an eGFR equation to a reference value. McNemar’s test with Yates correction was performed to compare the P_30_ values of two eGFR equations on the basis of paired proportions. For each eGFR equation, the agreement between the mGFR and eGFR values in the Chinese cohorts was examined by LOWESS curve fitting, as described by Inker et al.^4^ To analyze the correlation between the normalized abundances of individual metabolites and demographic/clinical characteristics (i.e., mGFR, age, body height, and body weight), Spearman’s rank correlation was performed. When comparing the normalized abundances of individual metabolites between the male and female CKD patients as well as between the CKD patients with severe nephropathy, the Mann–Whitney U test was performed. When controlling for confounding effects of age, sex, body height, body weight, and/or blood sample type (i.e., serum or plasma), partial correlation analysis was performed to examine the correlation between the natural logarithm-transformed normalized abundance of a metabolite and natural logarithm-transformed mGFR (ln(mGFR)). To handle the multiple hypothesis testing issue, all *P*-values were adjusted using the Benjamini–Hochberg method. For Discovery Group 1A and Group 1B, the percentile rank of each metabolite in each group was calculated according to correlation coefficients obtained from the partial correlation analyses. Only those metabolites that were negatively correlated with ln(mGFR) were included for the calculation of the percentile ranks. The metabolites were then ranked according to average values of percentile ranks calculated for Discovery Group 1A and Group 1B. The statistical analyses, bootstrapping and equation construction were performed using SPSS (version 27.0, IBM Corp., Armonk, NY). LOWESS curve fitting was performed using Peltier Tech Charts for Excel (version 4.0, Peltier Technical Services, Inc., Shrewsbury, MA).

**SUPPLEMENTAL RESULTS**

**Result S1. The percent Agreement within 30% of the mGFR (P_30_) for the eGFR Values Estimated by Old and New eGFR Equations for the Chinese CKD Patients**

For Chinese CKD patients, the P_30_ value of the eGFRcr(AS) equation was 69% (95% CI of 62% to 75%), which was significantly less than 80% (one proportion z-test, two-tailed, *P* = 0.0002), whereas the P_30_ value of the eGFRcr-cys(AS) equation was 79% (95% CI of 73% to 85%), which was significantly less than 85% (*P* = 0.025). It appeared that the P_30_ value of eGFRcr(ASR) (74%, 95% CI of 68% to 80%) was also less than 80% (*P* = 0.0526). Only the eGFRcr-cys(ASR) equation (82%, 95% CI of 76% to 88%) and the eGFRcys(AS) equation (84%, 95% CI of 79% to 89%) resulted in P_30_ values >80%, which were not significantly different from 85% (*P* = 0.311 and *P* = 0.846, respectively).

**Result S2. Discovery and Validation of Metabolite Markers for Estimating GFR – A Stringent Metabolomics Study Design Involving Serum and Plasma Samples from Two Hospitals**

The normalized abundances of 546 and 473 metabolites were significantly correlated with the mGFR (determined by the iohexol clearance rate) in Discovery Group 1A (serum samples from 33 CKD patients from Guangzhou) and Discovery Group 1B (plasma samples from 75 CKD patients from Macau), respectively (Spearman’s rank correlation, Benjamini–Hochberg (BH) adjusted *P* values <0.05, Table S4 and Figure 2). In Discovery Group 1A, 128 metabolites (23%) of the 546 metabolites were significantly associated with patient age, while in Discovery Group 1B, 338 metabolites (71%) of the 473 metabolites were significantly associated with patient sex, height, weight, and/or age in either group (BH adjusted *P* values <0.05) (Table S4 and Figure 2). After controlling for the confounding effects of age, sex, body height, and body weight, the logarithm-transformed normalized abundances of 296 metabolites (279 negatively correlated; 17 positively correlated) were significantly correlated with the logarithm-transformed mGFR in both discovery groups in the same direction (partial correlation, BH adjusted *P* values <0.05) (Table S4 and Figure 2).

To further avoid the discovery of false biomarkers, we compared the normalized abundances of 296 metabolites between 12 CKD patients with severe nephropathy (from Discovery Group 1B) and 10 healthy volunteers (Discovery Group 1C). Only those that were significantly different (Mann–Whitney test, BH adjusted *P* values <0.05) and had a fold change >2 in a consistent direction were retained as putative biomarkers (Table S4) to minimize false biomarkers.^9^ A total of 215 putative biomarkers remained (Table S5 and Figure 2).

Using the 79 CKD cases for independent validation, 212 (97.8%) of them were successfully validated (207 negatively and 5 positively correlated with the mGFR, Table S5, BH adjusted *P*-value <0.05). using the independent validation dataset (i.e., 79 CKD cases, Figure 2), which had not been used for biomarker discovery. Therefore, the final list of putative biomarkers contained 212 metabolites.

**Result S3. eGFR Equations without Creatinine, Cystatin C, Age and Sex**

Multiple linear regression was used to construct eGFR equations to relate the logarithm-transformed mGFR to the logarithm-transformed normalized abundances of the top 20 putative metabolite markers (including creatinine) in the discovery dataset, as described by the CKD-EPI research group.^4^ Only the statistically significant metabolite markers (*P-*values <0.05) were retained in the model. S-adenosylhomocysteine (*P* = 0.010), gluconate (*P* = 0.00006), N6-succinyladenosine (*P* = 3.5 x 10^-7^), and hydroxyasparagine (*P* = 0.00008) were retained in the final equation named CKD-msMET4a (Table 2). When constructing the eGFR equation with the exclusion of gluconate. S-adenosylhomocysteine (*P* = 0.011), gulonate (*P* = 0.001), N6-succinyladenosine (*P* = 3.8 x 10^-7^), and hydroxyasparagine (*P* = 0.002) were retained in the second equation named CKD-msMET4b (Table 2). Further inclusion of age, sex, height, weight, and/or logarithm-transformed serum/plasma CysC did not significantly improve the two equations (*P*-values >0.05).

**Result S4. Independent Validation of the CKD-msMET4a and CKD-msMET4b Equations**

The performance of the two equations was evaluated using the independent validation dataset (i.e., 79 CKD cases, Figure 2), which had not been used for biomarker discovery and equation construction, and compared with the CKD-EPI eGFR equations (Table 2). Concerning bias, precision, accuracy and GFR category agreement, the CKD-msMET4a and/or CKD-msMET4b equations appeared to be one of the best two equations although the statistical power was insufficient. The P_30_ values of both equations were 82% (95% CI of 73% to 91%), which were not significantly different from 85% (one proportion z-test, two-tailed, *P*-values = 0.297). Both P_30_ values were not significantly different from the P_30_ values of the current eGFRcr-cys(ASR) equation (McNemar's test, *P* = 0.8897 and *P* = 0.8802, respectively) but were significantly different from the P_30_ value of the new eGFRcr(AS) equation (*P* = 0.0336 and *P* = 0.0251, respectively). As expected, LOWESS curves revealed the overestimation of GFR in the range of 30 to 90 ml/min/1.73 m^2^ in the validation cases for the CKD-EPI creatinine-based equations (i.e., eGFRcr(ASR), eGFRcr(AS), eGFRcr-cys(ASR), and eGFRcr-cys(AS)) (Figure S1, C to F) but not for the eGFRcys(AS) equation (Figure S1G). Such an overestimation issue was also not obviously observed for the CKD-msMET4a and CKD-msMET4b equations (Figure 3, A and B). Our results suggest that the CKD-msMET4a and CKD-msMET4b equations provide a better estimation of the GFR than the new creatinine-based equations without race.

The CKD-msMET4a and CKD-msMET4b equations did not include any demographic characteristics as a predictor. It was important to examine whether there would be any difference when applying the two equations to the male or female CKD patients. The ANCOVA results showed that was no significant difference for GFR estimated by both the CKD-msMET4a equation (mean male-to-female ratio of eGFR = 0.94, 95% CI: 0.83 – 1.05, *P* = 0.276) and CKD-msMET4b equation (mean male-to-female ratio of eGFR = 0.92, 95% CI: 0.82 – 1.03, *P* = 0.152). It is worth noting that in the independent validation group the blood creatine levels were significantly higher in the male patients (mean male-to-female ratio of blood creatinine level = 1.37, 95% CI: 1.21 – 1.56, *P* = 3.4 × 10^-6^). This could serve as a positive control to reveal that absence of obvious difference in the GFRs estimated by the two equations was not due to sample selection bias.

**SUPPLEMENTAL DISCUSSION**

**Discussion S1. Comparison to the Designs of Previous Metabolomics Studies of CKD**

Since 2016, there have been various metabolomics studies of CKD. The majority of these studies attempted to identify metabolites for understanding the progression of CKD, inferring kidney function, or predicting poor outcome.^15,16,18,20,29-31,33,34^ Before the present study, there were only two metabolomics studies, Titan et al.’s study^35^ and Coresh et al.’s study^19^, aiming at metabolites for GFR estimation. In Titan et al.’s study^35^, two patient cohorts were involved. Similar to the present study, serum samples were collected from one cohort, while plasma samples were collected from another cohort. The eGFRcr-cys(ASR) equation was used to estimate patients’ GFRs. Eleven metabolites were found to be significantly correlated with the eGFR in both cohorts. The major limitation of Titan et al.’s study^35^ was that potential metabolite markers were identified through correlation analysis with the eGFR instead of the mGFR. Furthermore, the team did not attempt to build a new equation for estimating GFR on the basis of the potential metabolite markers. In Coresh et al.’s study^19^, two patient cohorts, the AASK cohort and the MESA cohort, were involved. Serum samples were collected from both cohorts. Common metabolite markers were identified from the two cohorts and prioritized according to the degrees of correlations with the mGFR. Fifteen metabolites were selected for quantification by targeted MS-based assays. The absolute values of the selected metabolites from the AASK cohort were used to construct equations for estimating the GFR by multiple linear regression (stepwise backward elimination). A demographic-free equation based on beta-pseudouridine, myo-inositol, acetyl-L-threonine, and tryptophan was developed. Data from the MESA cohort were used to validate the performance of the equation. Compared to Coresh et al.’s study^19^, the present study adopted a design with various improvements.

First, in Coresh et al.’s study^19^, metabolites were ranked by correlation with the mGFR. Those metabolites strongly correlated with the mGFR and with feasible, reliable, targeted assays were selected for equation construction. However, their correlation analyses had not been adjusted for potential confounding factors, such as age, sex and body weight. In the present study, we showed that a substantial number of the metabolites that were significantly correlated with the mGFR (Spearman rank correlation test without adjustment for demographic factors) were also significantly correlated with age, sex, body height and/or body weight. Then, we ranked our metabolites according to correlation coefficients obtained by partial correlation with the mGFR upon adjustment for age, sex, body height and body weight. Our approach should be more reliable for identifying metabolites with a robust correlation with the mGFR. Coresh et al.’s demographics-free equation involved acetyl-L-threonine, beta-pseudouridine, myo-inositol, and tryptophan.^19^ All four metabolites were present in the metabolomics profiles of the two Chinese CKD cohorts. However, according to the partial correlation results, myo-inositol was ranked No. 26, and acetyl-L-threonine was ranked No. 47.

Second, Coresh et al.’s study^19^ and the present study both used the UPLC–MS/MS-based Metabolon platform to obtain quantitative untargeted metabolomic profiles. Our recent study showed that UPLC–MS/MS-based metabolomic profiling is susceptible to the discovery of false biomarkers irrelevant to the true biomarkers even in the absence of systemic and systematic biases.^9^ One possible cause could be the matrix effect, which is characterized by coeluting substances' alteration of the signal response.^36^ Using a fold change of two as a filtering threshold could help minimize the identification of false biomarkers.^9^ In the present study, we compared the normalized abundances of metabolites between 12 CKD patients with severe nephropathy and 10 healthy volunteers. Only those metabolites that were significantly different between the two groups with a fold change >2 in a direction consistent with the partial correlation results were retained as putative biomarkers (Figure 2A). This filtering criterion was absent in Coresh et al.’s study^19^. In Coresh et al.’s study^19^, serum tryptophan was positively associated with the mGFR and was included as one of the four metabolite markers in the demographics-free equation. In the present study, tryptophan was also positively correlated with the mGFR in both Chinese cohorts. However, tryptophan was excluded as a putative biomarker because we only observed a fold change of 1.67 (severe nephropathy patients-to-healthy volunteers ratio = 0.60, Table S4). Moreover, importantly, serum/plasma tryptophan in healthy people can be reduced by dieting.^37,38^

Third, in Coresh et al.’s study^19^, the validation group (i.e., the MESA cohort) was not totally independent from the process of eGFR equation construction because the metabolites were first selected according to the correlation coefficients calculated for both the AASK cohort and MESA cohort. In contrast, in the present study, none of the cases used for independent validation were involved in the process of biomarker discovery and eGFR equation construction. Last but not least, different from the present study, only serum samples were used in Coresh et al.’s study^19^. As the metabolite contents of serum and plasma are different, the applicability of Coresh et al.’s findings^19^ to plasma samples remains unknown.

Despite the stringent study design, one of the limitations of our study was that patients with other diseases (e.g., diabetes, polycystic kidney disease, renal vasculitis) that could lead to kidney failure were not recruited. In the future studies, the identified putative metabolite markers and the CKD-msMET4a and CKD-msMET4b equations should be validated with patients suffering other diseases that could lead to kidney failure.

**Discussion S2. Accessibility to Measuring the Identified Metabolites in Clinical Practice**

In the present study, the Discovery HD4™ platform (Metabolon, Morrisville, NC, USA) was used to obtain the patients’ untargeted metabolite profiles. The Discovery HD4™ platform was launched in 2014. It is commercially available and has been successfully applied to various metabolomics studies.^10-12^ It is a well-established platform that allows unambiguous identification and precise quantification of metabolites in biological specimens. Furthermore, targeted LC-MS assays could be developed for absolute quantification of the identified putative biomarkers as described by Coresh et al.^19^ Last but not least, 18 of the top 20 putative biomarkers were previously shown to be inversely associated with kidney function (Table S6). Therefore, we strongly believe that the identified putative biomarkers could be easily measured in clinical practice.

**SUPPLEMENTAL TABLES**

Please refer to the Excel named “Supplemental Tables” for the Tables S1 to S7

Table S1. Demographic and clinical characteristics of the two Chinese cohorts in the present study.

Table S2. Comparison of blood creatinine levels and cystatin C levels between male and female CKD patients in the two Chinese cohorts in the present study.

Table S3. Demographic and clinical characteristics of the study participants in the study groups for biomarker discovery and equation construction (Group 1A, Group 1B & Group 1C) and those for independent validation (Group 2A & Group 2B).

Table S4. Summary of the correlations between the measured glomerular filtration rate (mGFR) and normalized intensities of blood metabolites identified by LC-MSMS, and the correlations between mGFR and demographic characteristics of chronic kidney disease (CKD) patients.

Table S5. Summary of partial correlation analyses results and comparisons between chronic kidney disease (CKD) patients with severe nephropathy and healthy volunteers of 215 putative metabolite markers.

Table S6. Summary of previous studies showing an inverse association between kidney function and the blood level of a metabolite marker.

Table S7. Comparison of blood creatinine levels, blood cystatin C levels and GFRs estimated by the CKD-msMET4a and CKD-msMET4b equations between male and female CKD patients in the independent validation group.

**SUPPLEMENTAL FIGURE**

**
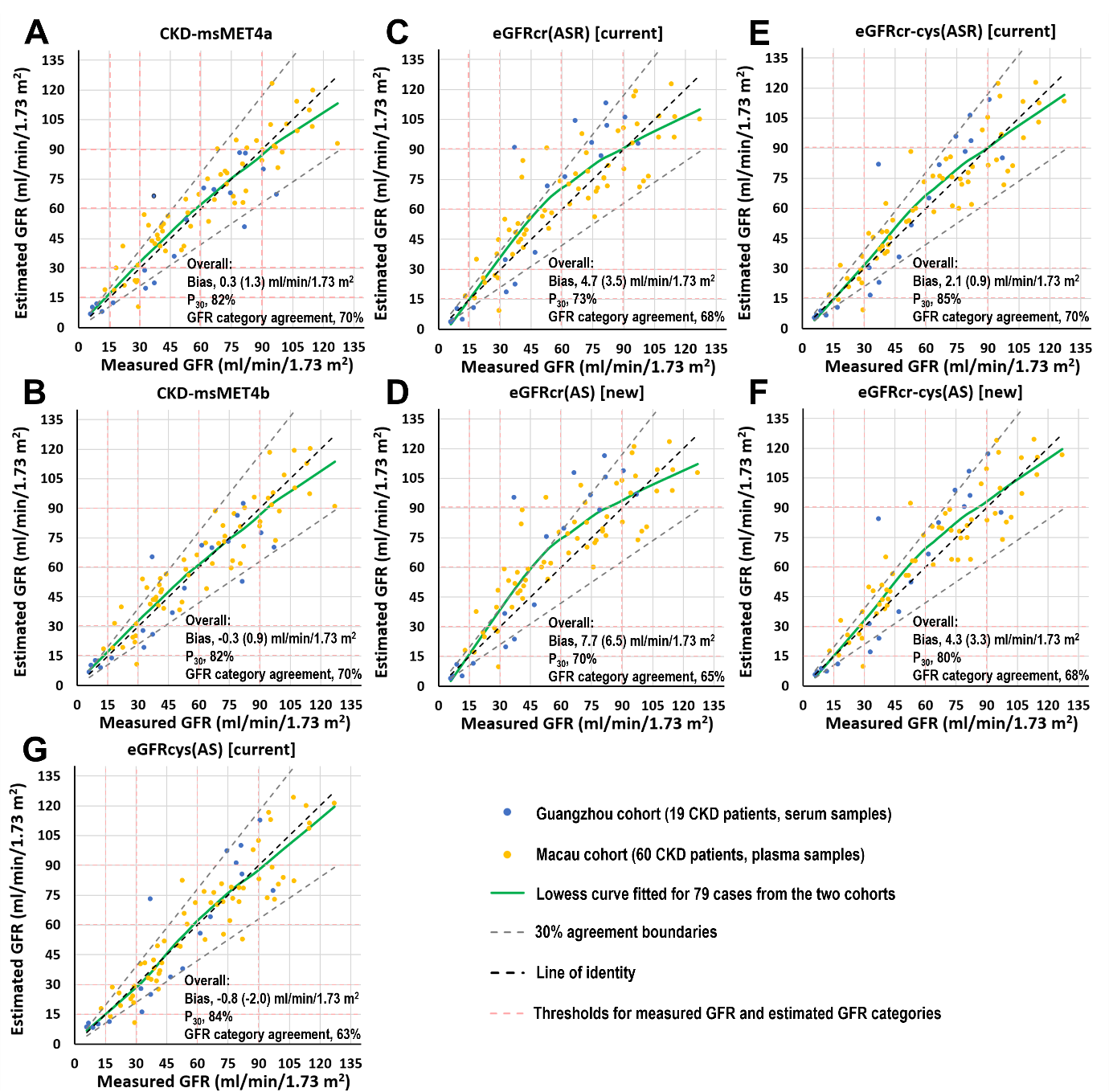
**

**Figure S1. Comparison of the mGFR and eGFR for the CKD-msMET4a equation, the CKD-msMET4b equation, and the CKD-EPI equations when applied to the independent validation cases.** The CKD-msMET4a equation was based on normalized abundances of serum/plasma hydroxyasparagine, S-adenosylhomocysteine, N6-succinyladenosine, and gluconate without demographic factors. The CKD-msMET4b equation was based on similar markers, except that gluconate was replaced with gulonate. The CKD-EPI equations are named according to the name provided by Inker et al.^4^ They are referred to by the filtration marker or markers (creatinine [eGFRcr], cystatin C [eGFRcys], or creatinine-cystatin C [eGFRcr-cys]) and the demographic factors (age, sex and race [ASR] or age and sex [AS]) that were used in their development. Data from the independent validation group comprising 19 patients (serum samples) from the Guangzhou cohort and 60 patients (plasma samples) from the Macau cohort are shown. Overall, we referred to the data of 79 patients from the two cohorts. Bias was defined as the mean difference (median difference) between the mGFR and the eGFR. A positive sign indicates overestimation of the mGFR, and a negative sign indicates underestimation of the mGFR. P_30_ is the percent agreement with 30% of the mGFR. GFR category agreement is the percent agreement between the mGFR and eGFR categories (<15, 15 to 29, 30 to 59, 60 to 89, ≥90 ml per minute per 1.73 m^2^). For each plot, the LOWESS curve was fitted using the data from 79 participants.

**SUPPLEMENTAL REFERENCES**

***(Numbering continues from the reference list in the manuscript)***

11. Suhre K, Shin SY, Petersen AK, et al. Human metabolic individuality in biomedical and pharmaceutical research. *Nature.* 2011;477(7362):54-60.

12. Shin SY, Fauman EB, Petersen AK, et al. An atlas of genetic influences on human blood metabolites. *Nat Genet.* 2014;46(6):543-550.

13. Dzúrik R, Lajdová I, Spustová V, Opatrný Jr K. Pseudouridine excretion in healthy subjects and its accumulation in renal failure. *Nephron.* 1992;61(1):64-67.

14. Niwa T, Takeda N, Yoshizumi H. RNA metabolism in uremic patients: accumulation of modified ribonucleosides in uremic serum. *Kidney Int.* 1998;53(6):1801-1806.

15. Sekula P, Goek ON, Quaye L, et al. A metabolome-wide association study of kidney function and disease in the general population. *J Am Soc Nephrol.* 2016;27(4):1175-1188.

16. Solini A, Manca ML, Penno G, Pugliese G, Cobb JE, Ferrannini E. Prediction of declining renal function and albuminuria in patients with type 2 diabetes by metabolomics. *J Clin Endocrinol Metab.* 2016;101(2):696-704.

17. Sekula P, Dettmer K, Vogl FC, et al. From discovery to translation: characterization of C-mannosyltryptophan and pseudouridine as markers of kidney function. *Sci Rep.* 2017;7(1):17400.

18. Niewczas MA, Mathew AV, Croall S, et al. Circulating modified metabolites and a risk of ESRD in patients with type 1 diabetes and chronic kidney disease. *Diabetes Care.* 2017;40(3):383-390.

19. Coresh J, Inker LA, Sang Y, et al. Metabolomic profiling to improve glomerular filtration rate estimation: a proof-of-concept study. *Nephrol Dial Transplant.* 2019;34(5):825-833.

20. Nierenberg JL, He J, Li C, et al. Novel associations between blood metabolites and kidney function among Bogalusa Heart Study and Multi-Ethnic Study of Atherosclerosis participants. *Metabolomics.* 2019;15(12):149.

21. Rhee EP, Waikar SS, Rebholz CM, et al. Variability of two metabolomic platforms in CKD. *Clin J Am Soc Nephrol.* 2019;14(1):40-48.

22. Cheng Y, Li Y, Benkowitz P, Lamina C, Köttgen A, Sekula P. The relationship between blood metabolites of the tryptophan pathway and kidney function: a bidirectional Mendelian randomization analysis. *Sci Rep.* 2020;10(1):12675.

23. Denburg MR, Xu Y, Abraham AG, et al. Metabolite biomarkers of CKD progression in children. *Clin J Am Soc Nephrol.* 2021;16(8):1178-1189.

24. Stam F, van Guldener C, ter Wee PM, et al. Homocysteine clearance and methylation flux rates in health and end-stage renal disease: association withS-adenosylhomocysteine. *Am J Physiol-Ren Physiol.* 2004;287(2):F215-F223.

25. Jabs K, Koury MJ, Dupont WD, Wagner C. Relationship between plasma S-adenosylhomocysteine concentration and glomerular filtration rate in children. *Metabolism.* 2006;55(2):252-257.

26. Valli A, Carrero JJ, Qureshi AR, et al. Elevated serum levels of S-adenosylhomocysteine, but not homocysteine, are associated with cardiovascular disease in stage 5 chronic kidney disease patients. *Clin Chim Acta.* 2008;395(1-2):106-110.

27. Zawada AM, Rogacev KS, Hummel B, et al. S-adenosylhomocysteine is associated with subclinical atherosclerosis and renal function in a cardiovascular low-risk population. *Atherosclerosis.* 2014;234(1):17-22.

28. Yu B, Zheng Y, Nettleton JA, Alexander D, Coresh J, Boerwinkle E. Serum metabolomic profiling and incident CKD among African Americans. *Clin J Am Soc Nephrol.* 2014;9(8):1410-1417.

29. Kimura T, Yasuda K, Yamamoto R, et al. Identification of biomarkers for development of end-stage kidney disease in chronic kidney disease by metabolomic profiling. *Sci Rep.* 2016;6:26138.

30. Luo S, Coresh J, Tin A, et al. Serum metabolomic alterations associated with proteinuria in CKD. *Clin J Am Soc Nephrol.* 2019;14(3):342-353.

31. Niewczas MA, Sirich TL, Mathew AV, et al. Uremic solutes and risk of end-stage renal disease in type 2 diabetes: metabolomic study. *Kidney Int.* 2014;85(5):1214-1224.

32. Chen S, Liu YH, Dai DP, et al. Using circulating O-sulfotyrosine in the differential diagnosis of acute kidney injury and chronic kidney disease. *BMC Nephrol.* 2021;22(1):66.

33. Hu JR, Coresh J, Inker LA, et al. Serum metabolites are associated with all-cause mortality in chronic kidney disease. *Kidney Int.* 2018;94(2):381-389.

34. Davies R. The metabolomic quest for a biomarker in chronic kidney disease. *Clin Kidney J.* 2018;11(5):694-703.

35. Titan SM, Venturini G, Padilha K, et al. Metabolites related to eGFR: evaluation of candidate molecules for GFR estimation using untargeted metabolomics. *Clin Chim Acta.* 2019;489:242-248.

36. Taylor PJ. Matrix effects: the Achilles heel of quantitative high-performance liquid chromatography–electrospray–tandem mass spectrometry. *Clin Biochem.* 2005;38(4):328-334.

37. Anderson IM, Parry-Billings M, Newsholme EA, Fairburn CG, Cowen PJ. Dieting reduces plasma tryptophan and alters brain 5-HT function in women. *Psychol Med.* 1990;20(4):785-791.

38. Attenburrow MJ, Williams C, Odontiadis J, et al. The effect of a nutritional source of tryptophan on dieting-induced changes in brain 5-HT function. *Psychol Med.* 2003;33(8):1381-1386.
